# Short-term volunteer health trips: aligning host community preferences and organizer practices

**DOI:** 10.1080/16549716.2017.1267957

**Published:** 2017-01-10

**Authors:** Michael D. Rozier, Judith N. Lasker, Bruce Compton

**Affiliations:** ^a^ Department of Health Management and Policy, University of Michigan, Ann Arbor, MI, USA; ^b^ Department of Sociology and Anthropology, Lehigh University, Bethlehem, PA, USA; ^c^ International Outreach, Catholic Health Association, St. Louis, MO, USA

**Keywords:** medical missions, capacity building, partnership/collaboration, voluntourism

## Abstract

**Background:** Short-term medical missions (STMMs) are quite common and largely understood to be a response to health needs in low-income countries. Yet most information about STMM practices is anecdotal. Even less is known about the preferences of in-country host communities regarding STMMs.

**Objective:** We aimed to gather enough quantitative and qualitative information from both STMM organizers and host community staff to compare dominant practices of organizers as well as preferences of host community staff. We use these data to discover differences between practices and preferences and suggest ways in which STMMs can be more responsive to the communities they serve.

**Methods:** Researchers gathered online survey responses from 334 STMM organizers and conducted interviews to determine existing practices. Similar methods were used to collect 49 online survey responses from, and conduct 75 interviews with, host community staff.

**Results:** Organizer practices and host community staff preferences are different in several areas. Organizers admit to minimal screening and preparation of volunteers whereas host staff have clear ideas of topics that should be covered in preparation, including culture and basic language skills. Organizers prioritize provision of clinical care during trips whereas host staff prioritize capacity building. Practices and preferences also differ in relation to the length of STMMs, the nature of the partnership itself, and the type of assessment and evaluation that is needed.

**Conclusions:** The large amount of data gathered for this study allows us to confidently say that organizer practices are often not aligned with host community staff preferences. Several concrete changes can be made to STMMs to bring practices more in line with the desires of the communities they serve.

## Background

Short-term volunteer trips have arisen as a major response to growing awareness of global health disparities. Every year, large numbers of Americans, Europeans, and people from other wealthy parts of the world travel to developing countries to participate in short-term programs intended to improve the health and well-being of people in poor communities [1]. Sometimes referred to as short-term medical missions (STMMs), such trips involve hundreds of thousands of people annually traveling from wealthier countries to poorer countries for a couple days to several weeks of health-related activities [2]. These are sponsored by a variety of organizations, including educational institutions, hospitals, faith-based organizations, other non-profits, and corporations. They vary in objectives, from specialized surgery to health education. They vary in length, from days to several weeks. And they vary in the ways and degree to which host partners are involved.

While many suggest the need for more information on STMMs, a recent systematic review found that nearly 95% of the articles on STMMs lacked significant data collection or evidence of outcomes [3]. One of the many gaps in knowledge established by this review of the literature notes the scant research on the preferences of host communities [4,5]. The current study, based on surveys and interviews of over 500 respondents, begins to fill the gap in the data by systematically comparing the preferences of host community staff and the practices of US-based STMM organizers. Divergence between actual and desired practices exists in multiple domains. We consider some explanations for the misalignment between what organizers do and what host community staff consider most valuable. Finally, we provide possible ways forward that align organizer practices and host preferences.

One consistent aspect of these trips, however, is the way that media outlets, including social media, are full of stories lauding the dedication of volunteers and their valuable impact in poor communities. Yet short-term international volunteer programs have increasingly been the target of criticism, with references to ‘drive-by humanitarianism’ [6], ‘slum tourism’ [7], and a new form of colonialism [7,8]. These criticisms focus on the hierarchical relationship between volunteers and host communities and on the self-serving character of much volunteering. Such criticisms often take philosophical or sociological approaches to STMMs, contending that the very premises upon which these trips are based is flawed.

Other criticisms of STMMs are much more practical. Some suggest trips cause harm to patients by exposing them to students who are gaining experience in medical care without adequate prior training [9,10]. Volunteers often provide care for free, which seems laudable, but often makes it difficult for local health workers to make a living even after volunteers leave because STMMs create an expectation among community memebers of free care. Lack of follow-up after brief medical and surgical interventions raises the real possibility of patients experiencing complications and side effects with no possibility for care [3,11,12]. Yet another concern is that the monies spent on flying volunteers around the world could be spent much more effectively in other ways, such as training local health workers.

While the debate about the value of volunteering continues, there is very little data to support either point of view. Both critics and supporters of STMMs often rely solely on anecdotes to bolster their particular position. Indeed, there is rarely any systematic evaluation of these programs to assess their value for host communities or for volunteers [13–16].

Most of the research on volunteering, both domestic and international, focuses on the motivations of volunteers and the benefits to volunteers [17,18]. Only recently have there been a few small studies that ask host country health program staff about their perceptions of the benefits and disadvantages of having volunteers [4,19,20]. These studies generally concur that some volunteers who make a commitment to the host country in a spirit of mutual respect can be helpful, but otherwise they considered the volunteers to be an expensive, time-consuming, and inefficient approach to health that can foster dependency and undermine local resources. In one study, Namibian health professionals were concerned with volunteers’ lack of cultural understanding of the country, resulting in offending people and making the visit ineffective [19].

We approached this study trying to avoid any preconceived position as to whether STMMs were valuable or damaging. Rather, we simply wanted to know what organizers are doing and what host community staff believe should be done.

## Methods

This paper combines results from three surveys and four sets of interviews conducted between 2012 and 2015 with people involved in STMMs, either as trip organizers in the US or as host community staff in several countries around the world (see Table 1).

**Table 1.** Summary of studies.

	Lehigh study		Catholic Health Association study	
Volunteer organizer	Survey	Interviews	Survey	Interviews
Year	2012	2012–2013	2014	2015
Respondents	*n* = 177	*n* = 27	*n* = 157	*n* = 18
Most represented destinations	Haiti, Honduras, India, Kenya, Uganda, Ecuador, Peru		Haiti, Guatemala, Mexico, Dominican Republic, Honduras, Peru, Kenya	
Host community		Interviews	Survey	Interviews
Year		2012–13	2015	2015
Respondents		*n* = 55	*n* = 14	*n* = 20
Most represented host countries		Ecuador, Ghana, Haiti, Niger	Haiti, Nigeria, Peru, Guatemala, Belize, Kenya, Nicaragua, Tanzania	

### Studies of STMM volunteer organizers

In 2012, a research team from Lehigh University contacted 611 organizations identified through an extensive Internet-based search for organizations based in the US that send volunteers abroad to do health-related work on a short-term basis (see [13] for details on the search methods). One hundred and seventy-seven individuals completed a survey using SurveyMonkey, including 89 from faith-based organizations, 26 from educational organizations, and 62 from other non-governmental organizations (NGOs). All respondents were actively organizing short-term international volunteer medical trips.

This survey was followed in 2012 and 2013 by in-person or telephone interviews with 27 officials of sponsor organizations in the US. Interview subjects constituted a convenience sample of organizers located all over the US and representing a variety of organizational types. Some had responded to the Lehigh survey and indicated they were willing to have a follow-up interview, and others were identified by word of mouth. Of the 27 interviewees, 2 work for corporations, 6 for educational institutions, 5 for faith-based organizations, and 14 for other NGOs.

In 2014, a second survey of STMM organizers in the US was designed by the three co-authors [21] based on the Lehigh survey and with many of the same closed-ended and open-ended questions. The Catholic Health Association (CHA), whose members operate over 600 hospitals in the US, sent a request to participate via email to all people known to be involved in international health projects at its member organizations. Many recipients forwarded the survey to colleagues in other organizations. The data in this study are based on the 157 respondents who had organized a short-term volunteer trip in the previous 5 years. Because of the anonymous nature of the Lehigh and CHA surveys, there is no way to know whether someone responded to both surveys, but none of the 611 organizations contacted as part of the Lehigh study were Catholic hospitals. All completed the survey through Qualtrics.

Eighteen individuals who indicated a willingness to be interviewed while responding to the CHA survey of organizers were subsequently interviewed over the telephone. The interview script, like the survey, was based on the surveys conducted in the Lehigh study. The purposive sampling strategy relied on the free-text responses in the survey and targeted those respondents who had provided particularly thoughtful reflections – either positive or negative – on their experience with STMMs. The majority worked with Catholic-affiliated organizations and there was no overlap with those who were interviewed for the Lehigh study.

### Studies of STMM host communities

For the Lehigh study, in-person interviews were carried out in 2012 and 2013 with 55 host community staff in Ecuador, Ghana, Haiti, and Niger. Using a referral-based sampling method, the interviewees included physicians, nurses, interpreters, drivers, community-based health workers, and administrative staff, all native to the countries where they were interviewed or from neighboring countries.

In 2015, a survey of host community staff was conducted by Accenture Development Partnerships under the direction of CHA. The survey used much of the same language as the survey of volunteer organizers and was distributed by CHA and its members via email to hospitals and clinics that receive short-term medical mission trips. Forty-nine individuals from 14 countries completed the survey [21].

The survey was followed by 20 interviews of host community staff. All 20 of the interviewees had responded to the CHA survey, indicating a willingness to be interviewed. Accenture selected an additional five interviewees as international experts in this area based on their reputation in the literature or among fellow practitioners. There was no overlap with those who were interviewed in the Lehigh study.

All surveys were analyzed using SPSS, Version 18 [22]. All interviews from the Lehigh University projects were audiotaped and transcribed. The project director (Lasker) developed a coding scheme in consultation with colleagues with expertise in qualitative methods. Three research assistants then coded several interviews together until consensus was reached. They subsequently coded all remaining transcripts under the supervision of the project director. The results were analyzed using Atlas-ti, Version 7 [23].

All survey and interview participation was completely voluntary and anonymous unless the participant chose to share identifying information. No compensation was provided to participants as study organizers determined participants expressed interest in the research even without incentives.

## Results

Organizer practices and host community preferences are described in greater detail in previously published literature [13,14]. Data from survey respondents who were volunteers on trips are also available in this literature. In this report, we bring together the data of trip decision-makers – the organizers and the host communities – and describe the contrasts between reported organizer practices and host country staff preferences in several of the domains covered in the studies (see Table 2).

**Table 2.** Volunteer organization practices and host staff preferences.

	Dominant practices of organizers	Dominant preferences of host community staff
Length of trip	1–2 weeks	3 weeks or longer
Selection of volunteers	Most applicants accepted; minimal screening	Should have skills, humility, and willingness to work and follow rules
Preparation of volunteers	Primarily travel information such as shots and packing	Should have preparation for language, culture, and work conditions
Nature of partnerships	Not all have partners; partners mostly subordinate in assisting and planning	Should have equality of decision-making, mutuality in relationship
Primary goals of trips	Direct provision of care	Capacity building
Needs assessment and evaluation	Done informally or not at all	Should be collaborative with host partner

### Length of trip

The vast majority of volunteer trips last 2 weeks or less, as reported by both organizers and host community staff.

In the CHA survey of organizers, almost 9 in 10 respondents reported that their *most recent* trip was 2 weeks or less (44% reported 1 week or less); in the Lehigh survey, organizers were asked about their trips *in general*. The most commonly chosen length (52%) was 2–3 weeks, followed by 1 week or less (38%). Organizers surveyed by CHA agreed that the ideal trip would be longer than the current length of most trips.

Host community staff working with volunteers voiced a strong consensus that trips should last longer than the current practice. The great majority considered visits of less than a week inadequate. Participants in the CHA survey of host organizations were divided between those who consider 8 days to 2 weeks ideal (39%) and those who prefer more than 2 weeks (37%). In a CHA interview, the chief medical officer in an African teaching hospital explained the preference for between 1 and 2 weeks: ‘1 week or less is a “recipe for trouble”. Patients don’t show up on time, autoclave won’t work, the whole trip is a waste. If 2-plus weeks, we get concerns on the care of the team itself and have security issues.’

Many host community staff commented that the shorter stays do not allow enough time to get to know the country and the work, limiting what a volunteer can accomplish. In the Lehigh interviews of host staff, there was a striking preference for visits of at least 3 or 4 weeks. An interviewee in Niger stated, ‘2 weeks we can do something. 3 weeks we can do something. 1 month we can do something. But 2 or 3 days, no.’

In both studies, some host community staff acknowledged that it may be impossible for volunteers to leave home for more than a week. Yet they also believe that for a visit to accomplish something, most volunteers have to spend time getting to know the work and the environment, and ideally they would stay for at least 2 or 3 weeks.

### Selection of volunteers

The majority of organizers reported minimal selectivity in choice of volunteers. In the CHA survey, 77% reported accepting more than three-quarters of all applicants; 41% accepted 95–100% of applicants. In the Lehigh survey, 52% accept more than three-quarters of applicants. Screening of volunteers ranges from minimal to a thorough evaluation of credentials and personal interview. As one of the Lehigh survey respondents on the minimal end explained, ‘We accept any one willing to come and work with us; we do not have any procedure.’

Host staff in both studies expressed a strong desire for choosing volunteers who have skills and humility. When asked what are the three most important qualities required of good volunteers, the top responses in the CHA host survey were ‘ability to work well with a team’, ‘willingness to learn from local community’, and ‘technical skills’. Similarly, when host staff in the Lehigh interviews considered the qualities of the best volunteers, they responded with a preference for those who are willing to work hard, have communication skills, and are adaptable, technically skilled, and humble and respectful. Ultimately, there is considerable overlap between organizers and hosts regarding the desirable qualities of a volunteer. The gap between them is found primarily in the lack of selectivity on the part of many organizers who do not assess these qualities and therefore do not use them as a basis for accepting or rejecting applicants.

### Preparation of volunteers

Organizers were also asked what type of information and/or orientation they provide. The majority (93% Lehigh; 98% CHA) provide information packets that include advice about travel, shots, and packing. Approximately 70% in each sample offer reading materials about the country to be visited. More than half have an in-person orientation program, although many of these occur upon arrival in the country. One in 10 in the Lehigh survey reported offering no orientation at all.

Despite organizer claims of preparation in certain areas, the CHA survey of host organizations revealed several areas in need of improvement. In the CHA survey of host organizations, participants were asked what volunteer qualities needed the most improvement. The top responses were ‘ability to train local staff to provide better care for patients’ (68%), ‘knowledge of local customs and culture’ (64%), and ‘willingness to learn from local community’ (50%).

Host community staff interviewed in both studies want volunteers to be prepared in advance of their arrival in language, culture, the nature of the projects to be carried out, and country conditions. For example, a Nigerien recommended, ‘I would speak about our culture; I would speak about the importance of physical and spiritual healing of the children.’ Several referred to the need for understanding local customs around dress and behavior. A Ghanaian employee suggested, ‘I would take the opportunity to tell them about the kind of environment they’re coming into. This hospital, for instance, the kind of inpatients we usually have, the kind of people they’re going to come in contact with, maybe possible problems they’d face.’ A Nigerien, for example, wanted to prepare volunteers for a variety of conditions they might not be familiar with: ‘They might be shocked about certain behaviors or certain illnesses, the temperature, the climate. The conditions under which people are living here.’

Including these diverse topics is not possible unless organizers require additional time from volunteers. In the CHA survey, over 40% of respondents said the in-person orientation lasted only 1–2 hours. Nearly 40% also responded that it should last at least half a day while over 25% said it should last one full day or longer.

### Involvement of an in-country partner

STMMs require partnerships between organizers in countries that send volunteers and host country staff that receive them. Yet, in the Lehigh survey, only 54% reported *always* having a regular in-country partner for their missions. An additional 18% *usually* do so. That means that almost 3 in 10 do not or only sometimes partner with existing organizations in the country. Among the organizers surveyed by CHA, 91% reported working with an in-country organization in their *most recent trip*.

In the CHA survey of organizers, respondents were asked about the role of the in-country partner. Partners most frequently provide logistical support (88%), assist volunteers to provide services (72%), and help define the goals of the trip (69%). Organizers indicated that on an ideal trip, partners would be more actively involved in defining goals and activities.

Host community staff agree with the goal of their being involved beyond logistics. In the CHA survey, host community staff were asked about their organization’s level of involvement in defining the goals and activities of the medical mission trips. One in four reported minimal or no involvement; the others said they were equally (27%) or primarily (29%) responsible. 8.3% indicated that they were 100% responsible. Yet all host participants in the CHA survey expressed a preference for equal involvement (54%), primary responsibility (27%), or 100% responsibility (13%) and 6% of respondents answered they didn't know who should set the goals of the trip. This is consistent with the interviews of host country staff in the Lehigh study, many of whom expressed strong preferences for more involvement in the planning and activities of the volunteer programs.

### Primary goals of trips

In the CHA survey of host community staff, 70% of respondents indicated that they wanted the STMM to focus more on training local staff than directly treating patients. Only 55% of respondents indicated that STMMs provided the opportunity for local doctors to shadow in primary care settings. The percentage was even lower in speciality care (53%), patient screening (32%), and the prescribing of medication (28%). Other areas of capacity building were no better. Forty-two percent of host community staff indicated receiving assistance in collecting and managing patient data. Only 20% of host community staff indicated receiving assistance with hospital leadership. These results should not be surprising given what organizers perceive to be the most important goals of STMMs.

When asked what the most important goals of the STMMs are, only 25% of organizers in the CHA survey and 22% of those in the Lehigh survey indicated that building capacity in host country medical facilities was among their most important goals. The most frequently included top goals in the CHA survey were improving access to medical or surgical care (73%), providing volunteers with an opportunity to serve (38%), and improving public health conditions (35%). The Lehigh survey produced similar priority areas. Given the perception of organizers as to the most important objectives of these trips, it should not be surprising that over two-thirds of host community staff would like to see more training of local health care providers.

### Monitoring and evaluation

Findings revealed a lack of needs assessment before trips and evaluation afterward. Fifty-five percent of respondents in the CHA survey of host community staff indicated that some type of needs assessment was conducted to help prioritize the goals for the trip; this means that nearly 45% of respondents did not participate in or were unaware of a needs assessment occurring.

With regard to evaluation, the Lehigh survey of organizers found that only one in four (27%) ever attempted to evaluate the impact of their activities on host communities. In the CHA survey of organizers, the most commonly cited evidence of success was an invitation to return to the same community. Less than 20% of organizers used evidence related to health outcomes.

On the other hand, nearly three-quarters of organizers from Lehigh’s survey evaluated the experience of the volunteers. The CHA survey found that debriefing of volunteers was done regularly. Interviews with organizers indicated a lack of attention to evaluation of impact on community health, with feedback being obtained mostly informally from conversations during the trip with host community staff or from anecdotes of patient improvements.

Over 75% of host organization staff from the CHA survey said that they have an opportunity to provide feedback to visiting organizations. However, while the ability to provide feedback is perceived by volunteer organizers to be there, interviews identified misalignments between US-based organizations and their international partners. For example, some host community leaders said that the fear of losing the partnership is a major obstacle to providing real feedback to the volunteer organizers; even when they do, they perceive that feedback is rarely incorporated into future planning efforts.

## Discussion

There are several possible explanations for the gaps between organizers and host community staff identified by this research. One explanation is that the goals of sponsoring organizations go beyond (and in some cases barely include) providing service to poor communities [24]. For example, the primary, if sometimes unstated, focus of an STMM might be the formation of the volunteers themselves [25,26]. This goal may include exposing volunteers to the poverty of developing countries in order to raise awareness or building relationships across countries. If this is the case, we believe organizers of STMMs should clearly state these objectives for all to see.

A less positive example is that many organizations arrange volunteer trips to advance their own reputations. Not much is known about the impact of STMMs on volunteer organizations, but studies from academic medicine indicate there are crosscutting motivations, including the development of a reputation or brand as well as the generation of new revenues [27,28]. STMMs attract students, employees, or church members to organizations, thus enhancing their public image and building a larger network of supporters of the organization in the US. For some it is about increasing income, either for profit or for supporting programs. These goals are consistent with promoting greater participation and can lead to greater attention to volunteer needs than to those of host communities. They can easily limit incentives to invest in screening and preparing volunteers, investing in long-term relationships with host community staff, and conducting rigorous needs assessments and evaluation programs.

It must also be said that STMMs are very complicated to organize. Enormous amounts of logistical details necessarily consume the attention of organizers. Placing host community needs at the top of the priority list, and recognizing that those needs are often not met well, will require a culture change among STMM organizers and host communities.

A final explanation can be found in the power differential between STMM organizers and host communities. Too many organizations that send volunteers do not have an ongoing relationship with host community staff, and those organizers that do usually control the power in that relationship. Organizers are endowed with financial and educational resources, and often with an attitude of superiority due to living in a wealthy nation. This has led to the critique that these trips are less volunteering than they are ‘voluntourism’ [29–31]. Host community staff are often dependent upon those resources to provide the basic care their communities require. Therefore, it is not surprising that sponsor organizations usually decide how the trips are to be organized and host organizations are often reluctant to challenge them.

There are several limitations of this research worth noting. First, representativeness and response rate for the surveys are difficult to establish. The Lehigh survey attempted to identify every US organization involved in STMMs, but many churches and other organizations that send occasional missions do not have web presences. The response rate was 29% of the researchers’ final list. The convenience sample also makes it impossible to establish a response rate for the CHA survey. Second, the research with host community staff was primarily carried out by Americans, perhaps creating a perception that the researchers were affiliated with STMM organizers or sponsoring organizations and thus muting criticism of STMMs. Finally, a third limitation concerns the generalizability of the results to STMMs based in countries outside of the US. Further research on programs originating in other countries is in order.

## Conclusion

Whether STMMs do more ill or more good remains an open question. In order to answer that question, all involved in these trips will have to begin taking measurement and evaluation more seriously. However, the data from these surveys and interviews do suggest a concerning divergence in the practices of organizers and the preferences of host community staff. Many host community staff (and quite a few US-based organizers) believe that changes to STMM practice could greatly enhance their impact.

In response to this and other research [13,32], CHA created a set of Guiding Principles [33] for work in international health. From the perspective of organizers, these changes include:

- Self-Assessment – ensuring motives for action are appropriate and all goals are honestly and clearly communicated.
- Needs Assessment – working at the invitation of a host community partner and ensuring a recent needs assessment has been conducted in the community.
- Asset Assessment – working with a host community partner to ensure local resources are known and used as far as possible.
- Planning and Preparation – organizers and host community staff must together determine clear goals, perhaps creating a Memorandum of Understanding.
- Selection and Preparation of Volunteers – selecting only those volunteers who will advance the goals of the trip and providing them a thorough orientation that includes cultural competence and capacity building.
- Implementation – ensuring the highest standards of care are followed and collaborating to build capacity with local organizations.
- Monitoring and Evaluation – setting aside some of the limited resources for assessment of real impact on the health of the community and making this information available to all, starting with the baseline from the original needs assessment.

Other resources are available [34–36] that provide better ways forward for STMMs and will assist those organizers interested in strengthening their programs. We also recommend a robust research agenda focused on monitoring and evaluation so that the real impact of STMMs on community health can be firmly established and the practices proven most effective incorporated into future missions.

## Paper context

Short-term international medical missions have fervent supporters and equally fervent critics, both often relying on anecdotal evidence. Using several hundred surveys and interviews with volunteer organizers and host communities we establish that the dominant practices of medical missions – length of trip, nature of partnerships, primary goals of trips, and more – are different than what host communities would prefer. Host community preferences must be better known and must take higher priority when organizing trips, even when this makes trips more challenging for organizers.

## References

[CIT0001] Lough BJ. (2013). International volunteering from the United States between 2004 and 2012.

[CIT0002] Lough BJ (2015). A decade of international volunteering from the United States, 2004 to 2014.

[CIT0003] Sykes K (2014). Short-term medical service trips: a systematic review of the evidence. Am J Public Health.

[CIT0004] Green T, Green H, Scandlyn J (2009). Perceptions of short-term medical volunteer work: a qualitative study in Guatemala. Global Health.

[CIT0005] Reeve ME, Groce NE, Persing JA (2004). An international surgical exchange program for children with cleft lip/cleft palate in Manaus, Brazil: patient and family expectations of outcome. J Craniofac Surg.

[CIT0006] Csoman K, Nagengast E, Briggs L (2012). Ethical predicaments of promoting student internships in Africa.

[CIT0007] Frenzel F, Koens K, Steinbrink M (2012). Slum tourism: poverty, power and ethics.

[CIT0008] Cohen MA, Küpçü MF, Khanna P (2009). The new colonialists [Internet].

[CIT0009] Crump JA, Sugarman J (2008). Ethical considerations for short-term experiences by trainees in global health. JAMA.

[CIT0010] Ackerman LK (2010). The ethics of short-term international health electives in developing countries. Ann Behav Sci Med Educ.

[CIT0011] Roldán JC, Pape HD, Koch H (2016). Ten-year cleft surgery in Nepal: achievements and lessons learned for better cleft care abroad. Plast Reconstr Surg Glob Open.

[CIT0012] Welling DR, Ryan JM, Burris DG (2010). Seven sins of humanitarian medicine. World J Surg.

[CIT0013] Lasker J (2016). Hoping to help; the promises and pitfalls of global health volunteering.

[CIT0014] Lasker J, Rozier M, Compton B (2014). Short-term medical mission trips: phase I research findings–practices & perspectives of U.S. partners.

[CIT0015] Maki J, Qualls M, White B (2008). Health impact assessment and short-term medical missions: a methods study to evaluate quality of care. BMC Health Serv Res.

[CIT0016] Martiniuk AL, Manouchehrian M, Negin JA (2012). Brain gains: a literature review of medical missions to low and middle-income countries. BMC Health Serv Res.

[CIT0017] Lindenmeier J (2008). Promoting volunteerism: effects of self-efficacy, advertisement-induced emotional arousal, perceived costs of volunteering, and message framing. Voluntas.

[CIT0018] Sherraden MS, Lough B, McBride AM (2008). Effects of international volunteering and service: individual and institutional predictors. Voluntas.

[CIT0019] Kraeker C, Chandler C (2013). We learn from them, they learn from us: global health experiences and host perceptions of visiting health care professionals. Acad Med.

[CIT0020] Laleman G, Kegels G, Marchal B (2007). The contribution of international health volunteers to the health workforce in Sub-Saharan Africa. Hum Resour Health.

[CIT0021] Catholic Health Association of the United States (2015). Short-term medical mission trips: phase II research findings–practices & perspectives of international partners.

[CIT0022] SPSS Inc (2009). PASW statistics for Windows, version 18.0.

[CIT0023] ATLAS.ti (1999). Version 7.

[CIT0024] Lasker J (2016). Global health volunteering; understanding organizational goals. Voluntas.

[CIT0025] Yao CA, Swanson J, McCullough M (2016). The medical mission and modern core competency training: a 10-year follow-up of resident experiences in global plastic surgery. Plast Reconstr Surg.

[CIT0026] Rovers J, Japs K, Truong E (2016). Motivations, barriers and ethical understandings of healthcare student volunteers on a medical service trip: a mixed methods study. BMC Med Educ.

[CIT0027] Merritt MG, Railey CJ, Levin SA (2008). Involvement abroad of U.S. academic health centers and major teaching hospitals: the developing landscape. Acad Med.

[CIT0028] Ackerly DC, Udayakumar K, Taber R (2011). Perspective: global medicine: opportunities and challenges for academic health science systems. Acade Med.

[CIT0029] Proehl J (2015). Risks of voluntourism. Emerg Nurs.

[CIT0030] McCall D, Iltis AS (2014). Health care voluntourism: addressing ethical concerns of undergraduate student participation in global health volunteer work. HEC Forum.

[CIT0031] Seymour B, Benzian H, Kalenderian E (2013). Voluntourism and global health: preparing dental students for responsible engagement in international programs. J Dent Educ.

[CIT0032] Lupton RD (2012). Toxic charity how the church hurts those they help and how to reverse it.

[CIT0033] Catholic Health Association of the United States (2016). Guiding principles for conducting international health activities. https://www.chausa.org/docs/default-source/international-outreach/cha_guidingprinciples_web.pdf?sfvrsn=0.

[CIT0034] Crump JA, Sugarman J, Working Group on Ethics Guidelines for Global Health (2010). Ethics and best practice guidelines for training experiences in global health. Am J Trop Med Hyg.

[CIT0035] Hartman E, Larsen MA (2015). Fair trade learning: a framework for ethical global partnerships. International service learning: engaging host communities.

[CIT0036] Stone GS, Olson K (2016). The ethics of medical volunteerism. Med Clin North Am.

